# Recognizing the Contributions of Advanced Practitioners to Oncology Care: Are Current Metrics Enough?

**DOI:** 10.6004/jadpro.2016.7.7.6

**Published:** 2016-11-01

**Authors:** Amanda W. Yopp, Holly M. Wall, Kena C. Miller

**Affiliations:** Takeda Pharmaceuticals International, Cambridge, Massachusetts

Oncology care has improved with the development of new treatment modalities that extend life expectancy. According to the American Cancer Society (ACS), there were more than 14 million Americans with a history of cancer in 2014, and the number of survivors is expected to increase to almost 19 million by 2024 (ACS, 2014). Now, more than ever, the burden on the health-care system is to provide excellent but cost-effective care.

Although the number of oncology patients and survivors is increasing rapidly, the supply of oncologists is predicted to grow at a much slower rate. Between 2005 and 2020, there is estimated to be a 14% increase in visit capacity due to new oncologists entering the workforce compared with a 48% increase in demand for oncologist visits (Erikson, Salsberg, Forte, Bruinooge, & Goldstein, 2007). This could result in a shortage of 9.4 to 15 million visits, or up to 4,080 oncologists (Erikson et al., 2007). Implementation of the Affordable Care Act has the potential to exacerbate this issue: The uninsured population decreased by 16.4 million from the time the Act took effect through May 2015 (U.S. Department of Health & Human Services, 2015), which could translate to more patients seeking oncology care. Although the increasing number of cancer survivors is an indication of remarkable advances in oncology, care for this burgeoning patient population will likely impose a tremendous burden on the health-care system.

Advanced practitioners (APs), including nurse practitioners (NPs) and physician assistants (PAs), are important resources in today’s evolving health-care landscape. As a part of physician-AP teams, not only do APs share the patient load, they also help to ensure greater patient satisfaction by providing nonbillable services before, during, and after treatment (Pickard, 2014). These nonbillable value-adding activities include educating patients, monitoring treatments, managing adverse events, coordinating referrals and outpatient services, preparing for and performing procedures, securing insurance authorizations, and handling complicated phone triage (Hinkel et al., 2010; Pickard, 2014). The collaborative physician-AP team approach can allow patients to receive cost-effective, high-quality care in a number of traditional and nontraditional settings (Hinkel et al., 2010; Martin-Misener et al., 2015; Moote, Nelson, Veltkamp, & Campbell, 2012; Nevidjon et al., 2010; Stanik-Hutt et al., 2013). Thus, APs can mitigate the physician/oncologist supply shortage while meeting the needs of oncology patients (Blakely & Cope, 2015; Hinkel et al., 2010).

## The Need to Measure Contribution of APs

The AP contribution to oncology practice is multifaceted and complex, and it can be challenging for APs to recognize and understand how their contributions are assessed within their practice. In oncology and other clinics, APs often spend considerable time on nonbillable value-adding services, which greatly impact patient outcomes (Hinkel et al., 2010; Kutzleb et al., 2015; Pickard, 2014). When APs work as part of the oncology team, job satisfaction and team productivity are likely to improve (Erikson et al., 2007). Potential economic benefits (Martin-Misener et al., 2015) and quality of care (Stanik-Hutt et al., 2013) provided by APs can have an important impact on the overall success and sustainability of the practice or the institution (Fineberg, 2012). Advanced practitioners should also have a clear understanding of their practice’s expectations for demonstrating contributions and how these contributions are evaluated.

**Productivity and Value**

Although the role of APs in oncology care is clear and has expanded, the value of their contributions is not always fully appreciated. In fact, even a clear understanding and awareness of terms used to determine contributions are lacking. Productivity and value can both be used to describe and measure contributions of APs to clinical practice, but there are distinct differences between the two.

Productivity is a quantitative measure of clinical services provided and is an important measure in all areas of health care. To be sustainable in the current health-care environment, health-care providers (HCPs), specifically, physicians, NPs, and PAs, must be able to demonstrate and maximize productivity (Pickard, 2014; Rhoads, Ferguson, & Langford, 2006). Gross billing, number of patients, and net revenue have been identified as simple benchmarks to be used to measure productivity (Pickard, 2014).

Value is a qualitative measure of HCP contributions related to the quality of care and patient satisfaction. Value-adding activities may not increase quantitative productivity but are important aspects of patient care and practice success. Assessment of patient outcomes and patient satisfaction (measured via surveys) can yield a good estimation of the value added to the practice by HCPs (Pickard, 2014; Rhoads et al., 2006).

Although easier to measure, productivity alone does not reflect an accurate picture of the AP’s role in patient care. Furthermore, no standard measures exist to quantify the value of the contributions made by APs in the oncology/clinical setting.

We conducted a survey to assess whether the contributions of APs were being measured and how they were being measured. The goal of this article is to present the results of our survey and to stimulate conversation about quantitative and qualitative measurements of these contributions by APs. It is meant to encourage exploration by APs into how their contributions could be assessed more accurately and holistically. An overview of metrics currently in use is presented. Tools APs can use to evaluate their work and what APs can do to encourage dialogue with their employers about their contributions to the practice and overall quality of patient care are discussed.

## Use of Metrics in Clinical Practice

Several metrics to measure the contributions of HCPs in clinical practice have been described in the literature.

**Relative Value Unit**

Relative value units (RVUs) are the measurement used in the resource-based relative value scale system, an HCP reimbursement system used by the Centers for Medicare & Medicaid Services since 1992 (Johnson & Newton, 2002; Wynn et al., 2015). In this system, each current procedural terminology (CPT) code is matched to a total RVU amount, which is based on the provider’s work (work RVU), practice expenses, and malpractice cost (Johnson & Newton, 2002; Pickard, 2014).

Work RVUs take into account the following component of HCPs’ work: technical skills; time; effort and judgment required to perform a service; and stress resulting from performing a service (because of potential risk to the patient; Pickard, 2014; National Health Policy Forum, 2015). Relative value units, particularly work RVUs, enable work to be standardized across caregivers and to determine bonuses or pay (Pickard, 2014). In addition, RVUs are used outside the Medicare program by both private payers and practices to compare providers and ensure the maximization of practice resources (Johnson & Newton, 2002; Rhoads et al., 2006).

Although RVUs can be used to standardize some aspects of patient care, their use to calculate HCP contributions has several limitations (Johnson & Newton, 2002; Pickard, 2014; Rhoads et al., 2006). Nonbillable activities crucial to patient care are not accounted for when using the RVU system (Johnson & Newton, 2002), and many of these services are commonly performed by APs. Additionally, "incident to" billing and "shared visits" can mask the productivity of APs. These commonplace practices result when a payer does not enroll an AP in its billing plans, thereby necessitating AP billing under the physician provider number, or when practices wish to maximize reimbursement (Pickard, 2014; Yang et al., 2014). Unless practices specifically track the contributions of APs in these situations, which could increase costs for the practice, these contributions will remain hidden.

**Nursing Hours per Patient Day**

The nursing hours per patient day (HPPD) metric was introduced in 2002 as a tool to determine nursing productivity and staffing needs (Kirby, 2015; Twigg, Duffield, Bremner, Rapley, & Finn, 2011). This method categorizes hospital wards on the basis of patient and ward characteristics, then assigns nursing HPPD to each (Twigg et al., 2011). A retrospective analysis of data from 3 hospitals over a 4-year period suggested that the minimum staffing levels defined by this method could improve patient outcomes (Twigg et al., 2011). This method is currently being used in the United States, as confirmed by AP respondents of our survey (see section on "Results of AP Survey"). An important next step is to gain a better understanding of how the HPPD metric is being used in practices to measure the productivity of APs specifically.

**Limitations of Productivity Measures**

Although RVUs and HPPD are widely used across disciplines, they do not capture all value-adding services that APs bring to oncology clinics (Gilbert & Sherry, 2016; Kirby, 2015; Ogunfiditimi, Takis, Paige, Wyman, & Marlow, 2013; Pickard, 2014; SurveyMonkey, 2015). Important questions remain about how these productivity measures impact patient care and the functionality of the medical practice, as well as whether they accurately reflect the total contribution of APs to the practice.

Recently, two groups examined whether and how the contributions of APs were being measured in their communities. Both studies identified a deficiency in this area. The tools they tested to better capture the total contributions of APs and the results of the two studies are summarized here.

*Self-Reported Productivity Tool*: In an attempt to measure the contributions of oncology APs, a self-reported productivity tool for NPs and PAs was developed to record time spent on revenue-generating and value-adding activities in 15 National Cancer Institute–designated comprehensive cancer centers (Hinkel et al., 2010). Productivity was assessed via an online survey and was based on the number of new and follow-up patients seen, but the survey did not measure the acuity of the patients or how much time was spent with each patient.

Analysis of the results revealed that APs conduct many more follow-up visits than new patient visits. The survey also showed that NPs and PAs in all specialties except medical oncology have similar patient loads. The authors considered their self-reported productivity tool a step toward development of AP productivity benchmarking and planned to refine the tool’s format to be more widely applicable and to incorporate a billing component (Hinkel et al., 2010).

*Metrics Card*: An AP committee at the Abramson Cancer Center identified several metrics that could be used to determine the contributions of APs in oncology practice (Gilbert & Sherry, 2016). From these metrics, a metrics card was generated; it measured performance based on financial impact, professional development, patient satisfaction, and quality indicators. The metrics card was tested in a pilot program by the head/neck and lung groups at Abramson Cancer Center, and the results were found to be accurately reflective of the contributions of APs. The metrics card continues to be refined, and the authors are working to establish benchmarks and an auditing system to ensure quality control. The metrics card approach has the potential to quantitatively measure the contributions of APs and should promote professional growth within the team and improve quality of care for patients (Gilbert & Sherry, 2016).

## Results of AP Survey

To better understand current contribution-assessment practices, we conducted a national survey of oncology APs and nurses. Participants were given a link to an online survey (SurveyMonkey, 2015), and responses were collected between November 2015 and January 2016. In addition to demographic information, participants were asked about contribution measurements and their work experiences. The survey had an approximately 10% response rate and was answered by 59 APs (80% of respondents) and 14 nurses (20% of respondents) from community, regional, and academic practices in 22 states. Only results obtained from APs are discussed in the sections below.

**Figure 1:**
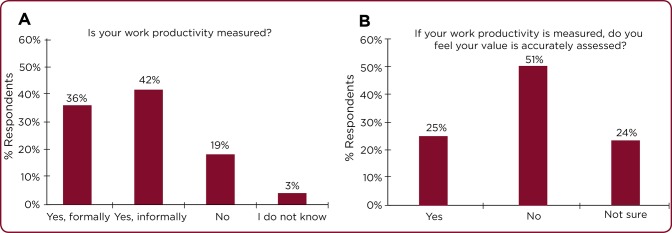
Productivity measurement (N = 59). Advanced practitioner (AP) respondents were asked (A) whether their productivity was measured and (B) whether it was assessed accurately.

**Figure 2:**
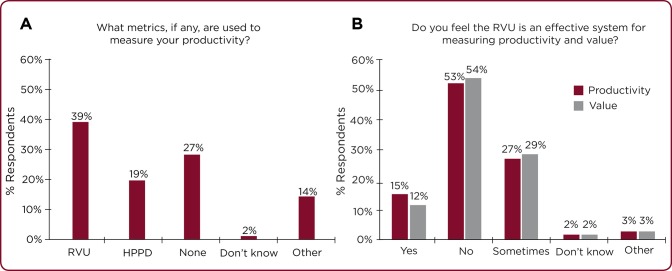
Measuring contributions (N = 59). (A) Metrics used to measure the productivity of advanced practitioners (APs). "Other" includes patient workload, annual group productivity, patient surveys, patient encounters, downstream revenue, referrals, laboratory payments, annual performance evaluations, and billable services and collections. (B) Opinion about the ability of RVUs to effectively measure the productivity and value of APs. “Other” includes nonfamiliarity with RVUs. (HPPD = hours per patient day; RVU = relative value unit).

Results indicated that AP productivity was either formally (36%) or informally (42%) measured for the majority of respondents (Figure 1A); however, only 25% of APs believed their productivity was being assessed accurately (Figure 1B). Among respondents, 39% and 19% reported using either RVUs or HPPD, respectively (Figure 2A); the majority of APs considered RVUs to be an ineffective measure of both productivity and value (Figure 2B). Other measures of productivity were patient workload, annual group productivity, patient encounters, patient surveys, downstream revenue, referrals, laboratory payments, annual performance evaluations, and billable services and collections.

**Figure 3:**
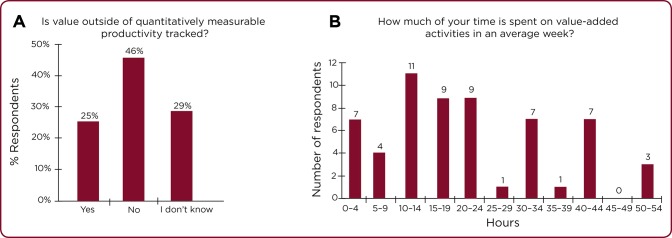
Value measurement (N = 59). (A) Advanced practitioner (AP) respondents were asked whether value outside of quantitatively measurable productivity was tracked. (B) Frequency distribution of the number of hours spent by APs on value-adding activities in an average week. The average number of hours per week was 19.8 hours. Value-adding activity examples defined in the survey were administrative projects; chemotherapy teaching; clarification of orders for pharmacy hospital staff; clinical research; coordination of care; dietary counseling; preparation of paperwork for the Family and Medical Leave Act, disability, and insurance; global visits for preoperative and postoperative care; hospital rounds/notes/discharge summary; patient education; symptom management via telephone; and triage.

Apart from quantitatively measurable productivity, value was not measured for many APs (46%), and another 29% did not know whether their value was being assessed (Figure 3A). This finding is despite the fact that respondents spent an average of 19.8 hours (range, 0–54 hours) weekly on value-adding activities that improve patient care and satisfaction (e.g., coordination of care, insurance, patient education, and symptom management; Figure 3B). On average, about half of APs’ time each week was spent on non–revenue-producing activities.

There were a few limitations to our survey. They include the lack of a glossary to clearly define productivity and value; the small sample size despite a broad target pool; and the fact that some questions did not include multiple-choice answers. Despite these limitations, our survey provides real-world evidence suggesting that neither value nor productivity of APs is being measured accurately.

## Implications of Survey Findings and Future Steps

The crux of the issue is whether APs can realistically gauge their value and contribution to a practice if the care they provide for their patients is not accurately assessed. Do APs feel valued by their practice, patients, and physicians? What significance does this have for overall job satisfaction and burnout rates? The answers to these questions may be important for retention of APs and should be used to initiate conversations between APs, practice administrators, and physicians to increase awareness of the contributions of APs to the practice.

Although a few metrics are currently being used to determine productivity, the imperative, nonbillable, value-adding services typically provided by APs should be specifically monitored to create a complete picture of the total care being provided. In the current landscape of oncology care, in which the contributions of each member of the health-care team must be maximized to maintain an effective and solvent practice, APs must proactively evaluate the metrics used to determine their contributions to clinical practice. If their contributions are not being monitored or are being monitored insufficiently, we recommend, as others have suggested previously, that APs consider tracking and recording their work activity and the time it takes to complete tasks, especially nonbillable services (Table). Patient satisfaction surveys and hospital admission data should be reviewed for objective measures of AP contributions. Furthermore, APs should also consider asking the practice to review productivity of the team before and after they joined the practice to raise awareness of their contributions. Prioritizing the services that are most important to the practice can help APs learn where to focus their productivity and performance efforts. These strategies can help raise awareness about the total contribution of APs to their practices (Table).

**Table:**
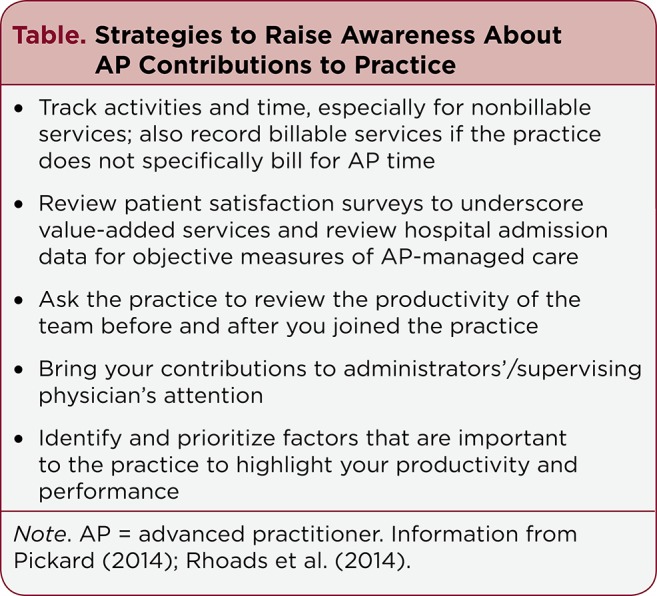
Strategies to Raise Awareness About AP Contributions to Practice

## Conclusion

Advanced practitioners are poised to fill the gap in care generated by the anticipated shortage of oncologists (Blakely & Cope, 2015). The value-adding services provided by APs are critical to oncology care, and the consistent delivery of these services is important for the success of the practice and for satisfactory patient experiences. Encouraging a better definition and understanding of AP value from the perspective of the practice, the physicians, and the patients is critical to ensuring that APs continue to contribute and thrive in the oncology arena. Advanced practitioners can do this by both advocating for patients and demonstrating how to be resourceful providers and part of the solution for quality, cost-effective oncology care. Additionally, APs need to be savvy regarding the business aspect of oncology. They must ensure that their contributions to the complex care of oncology patients are recognized, valued, and rewarded appropriately.

**Acknowledgments**

The authors would like to thank J. Kelly Buis, RN, MSN, ANP, for content review during manuscript development.
